# Urban Moveability and physical activity in children: longitudinal results from the IDEFICS and I.Family cohort

**DOI:** 10.1186/s12966-019-0886-2

**Published:** 2019-12-11

**Authors:** Christoph Buck, Gabriele Eiben, Fabio Lauria, Kenn Konstabel, Angie Page, Wolfgang Ahrens, Iris Pigeot, on behalf of the IDEFICS and the I.Family consortia

**Affiliations:** 1 Leibniz Institute for Prevention Research and Epidemiology – BIPS, Achterstraße 30, 28359 Bremen, Germany; 2 Section for Epidemiology and Social Medicine, Institute of Medicine, Sahlgrenska Academy, University of Gothenburg, Gothenburg, Sweden; 3 Department of Biomedicine and Public Health, School of Health and Education, University of Skövde, Skövde, Sweden; 4 National Research Council, Institute of Food Sciences, Avellino, Italy; 5 National Institute for Health Development, Tallinn, Estonia; 6 Centre for Exercise, Nutrition & Health Sciences, School for Policy Studies, Bristol, UK; 7 NIHR Bristol Biomedical Research Centre, University Hospitals Bristol NHS Foundation Trust and University of Bristol, Bristol, UK; 8 Faculty of Mathematics/Computer Science, University of Bremen, Bremen, Germany

**Keywords:** Accelerometer, Built environment, Childhood obesity, European children’s cohort, Physical activity, Walkability

## Abstract

**Background:**

Physical activity (PA) is one of the major protective behaviours to prevent non-communicable diseases. Positive effects of the built environment on PA are well investigated, although evidence of this association is mostly based on cross-sectional studies. The present study aims to investigate the longitudinal effects of built environment characteristics in terms of a moveability index on PA of children in their transition phase to adolescence using data of the IDEFICS/I.Family cohort.

**Methods:**

We used data on 3394 accelerometer measurements of 2488 children and adolescents aged 3 to 15 years old from survey centres of three countries, Germany, Italy, and Sweden, who participated in up to three surveys over 6 years. In network-dependent home neighbourhoods, a moveability index was calculated based on residential density, land use mix, street connectivity, availability of public transport and public open spaces such as green spaces and public playgrounds in order to quantify opportunities for PA of children and adolescents. Linear trajectories of light PA (LPA) and moderate-to-vigorous PA (MVPA) were estimated using linear mixed models accounting for repeated measurements nested within individuals. Least squares means were estimated to quantify differences in trajectories over age.

**Results:**

LPA and MVPA declined annually with age by approximately 20 min/day and 2 min/day respectively. In girls, the moveability index showed a consistent significantly positive effect on MVPA ($\hat{\beta}$ = 2.14, 95% CI: (0.11; 4.16)) for all ages, while in boys the index significantly lessened the decline in LPA with age for each year. ($\hat{\beta}$ = 2.68, 95% CI: (0.46; 4.90)).

Availability of public open spaces was more relevant for MVPA in girls and LPA in boys during childhood, whereas in adolescence, residential density and intersection density became more important.

**Conclusion:**

Built environment characteristics are important determinants of PA and were found to have a supportive effect that ameliorates the decline in PA during the transition phase from childhood to adolescence. In childhood environmental support for leisure time PA through public open spaces was found to be the most protective factor whereas in adolescence the positive influence of street connectivity and residential density was most supportive of physical activity.

## Background

Physical activity (PA) is one of the major protective behaviours to prevent adult non-communicable diseases such as cardiovascular diseases, diabetes, and obesity [1, 2]. Besides leisure time activities, evidence suggests that participation in active travel leads to higher levels of overall PA, particularly in children [3, 4] and substantially reduces the risk for cardiovascular diseases in later life [5].

Supporting effects of the urban built environment on PA are well investigated and positive effects of neighbourhood walkability on active travel particularly in children have been shown [6, 7]. Moreover, opportunities for leisure time PA in the urban neighbourhood of children were found to be positively associated with moderate-to-vigorous PA (MVPA) [8, 9]. However, evidence of the association between the built environment and PA is mostly based on cross-sectional studies [10, 11]. Up to now longitudinal studies are rarely conducted, but are required to investigate the causal relationship between the built environment and PA especially in children and adolescents with less evidence in an age group that undergoes vast changes [6, 12].

Walkability characteristics such as built environment characteristics of the street network and the urban neighbourhood area positively associated with walking for transport and leisure. Recent longitudinal studies showed that positive changes these characteristics can positively influence PA [9] and especially active travel [13] in adults. For instance, the RESIDE study [13, 14] investigated neighbourhood walkability and active travel in Australian residents before and after residential relocation based on four surveys. Longitudinal analyses revealed positive changes in walking frequency after participants relocated into more walkable areas. Moreover, transport-related walking decreased, but with improved access to transport-related and to recreational destinations higher frequencies of transport-related walking and recreational walking were found, respectively [13]. Overall, walkability measures such as land use mix, street connectivity and local access to public transport stops are suggested as important determinants of walking on the population level [14].

With regard to habitual PA in children, destinations for leisure time PA such as playgrounds or parks are important to consider alongside transport and school-related measures. As part of the IDEFICS study (Identification and prevention of dietary and lifestyle-induced health effects in children and infants) [15], a moveability index was developed based on the walkability concept that allowed a broader assessment of opportunities for PA in the urban environment of children particularly including designed public spaces for leisure time activities [16].

Schipperijn et al. (2015) [9] introduced a moveability index in a Danish sample of the European Youth Hearth Study that included a baseline survey and a six-year follow up. Results of this study revealed a positive cross-sectional association between urban moveability and PA. An increase in the moveability index that was observed in participants 6 years later after relocation in Denmark was associated with a reduced decrease in accelerometer-based PA in females.

In children and adolescents, PA strongly declines from childhood to adolescence. For example, Ortega et al. [17] found a yearly decline in MVPA of about 1 to 2.5 min per day from childhood to adolescence for boys and girls, respectively. Moreover, Farooq et al. [18] identified that the decline already started early at the age of 7 years, emphasising the importance of investigating the longitudinal effects across the childhood to adolescent period.

In a previous cross-sectional analysis of the IDEFICS study the moveability index and particularly the availability of public open spaces was positively related to objectively measured MVPA in school children [8]. However, longitudinal evidence regarding the effect of the built environment on physical activity particularly in children and adolescents is still lacking. Thus, the present study aims to investigate the longitudinal effects of urban moveability in relation to opportunities for both active travel and leisure time PA, and physical activity intensity of children and adolescents using data from a large prospective cohort of European children.

## Methods

### Study data

The population-based IDEFICS study, was conducted from 2006 to 2012 to investigate lifestyle-related diseases in European children and infants from eight countries (Belgium, Cyprus, Estonia, Germany, Hungary, Italy, Spain, and Sweden) [15]. The baseline survey (T0) took place between September 2007 and June 2008 including 16,229 2- to 9.9-year-old children [15]. The first follow-up survey was conducted 2 years later from September 2010 to May 2011, where 11,041 children aged 4 to 11.9 years participated in the follow-up examination (T1) and 2555 children were newly recruited [19]. In addition, a second follow-up (T2) was conducted that only assessed the penetration of the intervention messages by mail and that did not comprise the survey protocol of T0 and T1. Participants of the IDEFICS study (T0/T1) were re-invited to participate in the I.Family study for an enhanced third follow-up (T3) in 2013/ 2014 where 7105 children, their siblings and parents provided information based on an extended survey protocol [20] aiming to investigate entire families. In each country, the participating centres obtained ethical approval from the local ethics committees. Parents provided written informed consent for all examinations. Each child was informed orally about the measurements by field workers and asked for his/her consent immediately before the examination. The Pan-European IDEFICS / I.Family children cohort is registered under ISRCTN62310987.

The present analysis is based on data from baseline and follow-up surveys of the IDEFICS study as well as the I.Family study (T3) from seven study regions in three countries. We considered *N* =6185 observations of *n* =3287 children and adolescents who participated in the IDEFICS/I.Family cohort and wore an accelerometer device in at least one of the surveys (*N*_*T*0_ =2934, *N*_*T*1_ =1933, *N*_*T*3_ =1318). Environmental variables could not be calculated for *N* =1968 observations of children who did not directly live within the study area. Further, *N* =823 had to be excluded due to invalid or unavailable accelerometer measurements, leaving *N* =3394 observations of *n* =2488 participants. Most of the participants (*n*_1_ =1685) provided one valid observation, while two observations were provided by *n*_2_ =700, and three observations by *n*_3_ =103 participants. In this sample, only three variables had a small number of item missings, i.e. ISCED: 3.1%, safety concerns: 5.8%, and sports club membership: 6%, for which we included a missing category, each.

#### Physical activity

Habitual PA was assessed using Actigraph accelerometers (Actigraph,LLC, Pensacola, FL, USA). In IDEFICS (T0 and T1), either ActiTrainer or GT1M monitors were used, while in I.Family either GT1M or GT3x + devices were used. Participants were asked to wear the accelerometers for at least 3 days (including 1 weekend day) at T0 and T1 and for 7 days at T3. Accelerometers were mounted on the right hip during waking hours of each child using an elastic belt adjusted to ensure close contact with the body.

Details on processing of accelerometer data in the IDEFICS study as well as first descriptive results of accelerometer data of the IDEFICS study can be found in Konstabel et al. [21]. Valid measurements were defined as recording more than 360 min of at least one weekday and one weekend day after exclusion of non-wear time according to Choi et al. [22]. Nonwear time was identified using a 60 min. Window for each epoch to detect 30 min consecutive zero counts allowing breaks of 2 min of non-zeros. The threshold for valid measurements of 360 min. at least for at least one weekday and one weekend day was chosen as a trade-off between accuracy and sample size and is discussed in Konstabel et al. [21].

Before assigning intensity ranges, we here used a penalized expectile regression to smoothen the accelerometer counts that has been recently proposed in Wirsik et al. [23]. This method is able to identify underlying activity patterns similar to hidden Markov models (HMM) that were also proposed to improve modelling of accelerometer data. The penalized expectile regression was compared with the commonly used cut-off point methods and HMMs based on labeled data and outperformed the latter [23]. MVPA and LPA in minutes per day were then derived based on Evenson cut-off points for smoothed counts per minute (light: 104–2295, moderate: 2296–4011 cpm, vigorous: > 4011 cpm) [24].

BMI was calculated based on objectively measured height and weight that were assessed to the nearest 0.1 cm and 0.1 kg, respectively [19]. Age- and sex-specific BMI z-scores and categories for overweight and obesity were derived according to the extended IOTF criteria [25].

#### Covariables

Season of assessment was categorized as spring/summer if the accelerometer device was worn between March and September, and as autumn/winter, if assessment took place between October and February.

Education and qualification of parents were classified according to the International Standard Classification of Education (ISCED) [26]. We collapsed ISCED-levels into three categories, i.e. low (lower secondary education and less), medium (upper and post-secondary education), and high (tertiary education). We further added a category for missing values in ISCED-levels.

Parents were asked to respond to statements regarding safety concerns, i.e. “I restrict my child’s outdoor activities for safety reasons” and “I don’t like to let my child walk/cycle to kindergarten, pre-school or school for safety reasons” on a four-point Likert scale, i.e. disagree, moderately disagree, moderately agree, and agree. While both statements were part of the first two surveys (T0, T1), only the latter was included in the third follow up survey (T3). Agreement or strong agreement to any of the two statements was categorized as having safety concerns, while strong disagreement and disagreement was categorized as no concern.

Sports club membership (yes/no) was reported by parents for baseline and follow up (T0, T1). In T3 this was proxy reported by parents if the child was younger than 12 years, or self-reported, if the child was older than 12 years.

### Spatial data

In seven different study regions of three countries, geographical data were collected and processed to objectively assess built environment characteristics by means of a moveability index, i.e. Germany (Delmenhorst and Wilhelmshaven), Italy (Avellino, Atripalda, Mercogliano), and Sweden (Partille and Mölndal), using a geographical information system (GIS) (ESRI 2011. ArcGIS Desktop: Release 10.2 Redlands, CA: Environmental Systems Research Institute). Geographical data were processed to calculate the moveability index based on administrative data as well as open source databases.

The moveability index is an extension of the walkability index [27, 28] and quantifies opportunities for PA, in particular for active travel and leisure time PA, in the home neighbourhood of children. This index showed a positive association with MVPA in children based on cross-sectional IDEFICS data from one German study region [8].

In all study regions administrative data were provided by the land registry office of the local municipality or the federal state. Land use data were provided as adjacent polygons and condensed with regard to six different types including residential, commercial, industrial & agricultural, recreational, and miscellaneous. Residential density was obtained on district and subdistrict level. Geographic line data of the footpath network were obtained from the OpenStreetMap project (OSM) (www.openstreetmap.org – Open Data Commons Open Database License (ODbL)) and validated using administrative data. In all study regions a footpath network was built to calculate service areas and to derive intersections as point data. Bus stops and recreational facilities, i.e. playgrounds and parks, were digitally processed based on available maps and lists provided by the public transport companies and the civil service for green space of the municipalities [29].

### Home neighbourhoods

Addresses of participants were geocoded for each survey to derive network-dependent home neighbourhoods. We further accounted for residential relocation which, however, was not observed in participating children who provided two or three observations over time. If children relocated after participating first in either the baseline survey or the first follow-up and the new residential location was outside of the study areas this led to exclusion in the environmental analysis for the following surveys. Especially in the German study regions, it was not permissible to use the exact address coordinates to calculate individual home neighbourhoods due to data protection requirements. Therefore, we carried out spatial blurring based on a Gaussian error that was inversely proportional to the underlying residential density and conducted a simulation study, where spatial blurring shifted original coordinates by approximately 50 to 100 m in densely-populated areas induced only small differences in moveability measures [30]. We conducted the network analyses using the *network analyst* in *ArcGIS 10.2* and calculated the spatial blurring in *R 3.4.3* [31] using the *rnorm* function. Individual-level home neighbourhoods were defined based on network-dependent areas around the place of residence using a distance of 1250 m that was chosen based on previous research [30].

### Moveability index

The moveability index consists of the following five main components:

#### Residential density

Residential density, i.e. number of residents per area, was provided in districts or subdistricts of the considered study regions. For each home neighbourhood residential density was then derived as a weighted mean considering the size of the fraction of districts overlapping the home neighbourhood.

#### Land use mix

Percentages of land use types, i.e. residential, commercial, industrial & agricultural, recreational, and miscellaneous, in each network-dependent neighbourhood were derived to calculate land use mix based on the entropy formula [27].

#### Point characteristics

Point characteristics such as intersections, public transit stations and public open spaces were assessed using an anisotropic kernel intensity measure that provides consistent results over varying sizes of the home neighbourhood and tends to reduce bias through scaling and zoning [30]. This way, intersection density, i.e. street connectivity, as well as availability of public transport and public open spaces were calculated as mean intensity per home neighbourhood.

In order to compare opportunities for PA in each region instead of comparing the moveability between countries, z-scores were calculated separately for each region using the corresponding mean and standard deviation (SD) of the moveability index and its components, respectively. We further used the z-scores to dichotomise the moveability index and environmental variables into high (z-score ≥ 0) and low (z-score < 0). Spatial analyses were conducted using the *spatstat*-package [32] in *R 3.4.3* [31].

### Statistical analyses

Descriptive statistics, i.e. percentage or mean (SD) and range, of outcome, exposure variables and covariables were calculated based on the first examination of each of the *n* =2488 participants.

Age-dependent trajectories of MVPA and LPA were estimated using linear mixed models including two levels (repeated measurements nested within individuals) that allow to model different intercepts and age effects, i.e. these models allow study subjects to have their own trajectory over time, where individual trajectories for participants providing only one observation are calculated using supplement information by estimated population level trajectories. These models can easily handle unbalanced data with varying numbers of repeated measurements per subject, as well as subjects measured at different ages. Moreover, these models allow for change in scale and variance of the outcome measurements over time [33].

For each outcome variable, i.e. MVPA, and LPA, six models were estimated to investigate the effect of the moveability index as well as its five components on PA intensities with age. The model included a random intercept and random linear slope for age. Further, repeated measurements were taken into account by means of a random effect on the residual side. For each environmental variable a fixed effect as well as an interaction effect with age was included to model the effect of the built environment on MVPA and LPA over age. All models were adjusted for age (centred at 8 years), maximum ISCED level of both parents, parental safety concerns, sports club membership, valid wear time and season of accelerometer measurements, as well as study region.

In addition, all models were estimated stratified by sex, to investigate the effect of environmental variables to take into account the well-documented differences in PA intensities between girls and boys.

Estimated linear trajectories across age were depicted for high moveable and low moveable home neighbourhoods in boys and girls. Differences in these trajectories were quantified based on least square means (LSM) and 95% CIs that were calculated in each model for chosen age values, i.e. 4, 6, 8, 10, 12 and 14 years covering the age-range of our analysis.

We conducted sensitivity analyses by estimating linear trajectories over age similarly as described above using a reduced study sample of 1709 observations of 803 participants by only including children and adolescents who provided at least two measurements.

Statistical analyses were conducted in *SAS 9.3* (SAS Institute Inc., Cary, North Carolina, USA) and mixed models were estimated using the *glimmix* procedure. All results are presented at a significance level of α = 0.05 without adjusting for multiple testing.

## Results

Study participants’ characteristics are presented in Table 1. Overall, mean age was 7.5 years and 51.1% of the study participants in our sample were boys. Average MVPA was 48.7 min per day, which was higher in boys (53.8 min/day) than in girls (42.4 min/day). On average, children and adolescents had 293 min of LPA per day and about 20% were overweight or obese with no substantial difference between boys and girls. Environmental factors showed an average residential density of 2200 residents per km^2^ and on average 4.8 public spaces and 3.4 transit stations within the home neighbourhood (Table 1).

**Table 1:** Study characteristics, i.e. mean (M), standard deviation (SD) and range or sample size (*N*) and percentage (%) of *n* = 2488 children and adolescents based on the first examination in the IDEFICS/I.Family cohort of each child

	All (*n* = 2488)			Male (*n* = 1270)			Female (*n* = 1218)		
	Mean	(SD)	Range	Mean	(SD)	Range	Mean	(SD)	Range
Light PA (min / day)	293	(83.2)	(5.5–746.9)	292	(82.2)	(6.5–571.8)	293	(84.2)	(5.5–746.9)
MVPA (min / day)	48.7	(24.4)	(0–199.5)	53.8	(25.3)	(0–194)	42.4	(22.2)	(0–199.5)
Moveability index	0.04	(3.5)	(−13.5–10.8)	0.0	(3.4)	(− 10.7–8.7)	0.1	(3.5)	(− 12.9–10.8)
Public open space density	4.8	(4.1)	(0–18.3)	4.7	(4.1)	(0.04–16.7)	4.9	(4.1)	(0–18.3)
Residential density	2.2	(1.3)	(0–6.8)	2.2	(1.3)	(0–6.7)	2.2	(1.3)	(0–6.8)
Land use mix	0.72	(0.14)	(0.01–0.99)	0.72	(0.15)	(0.01–0.99)	0.72	(0.15)	(0.01–0.99)
Intersection density	7.7	(3.8)	(0.1–24.7)	7.7	(3.8)	(0–21.2)	7.8	(3.8)	(0.14–24.7)
Public transport density	3.4	(1.7)	(0–6.5)	3.4	(1.7)	(0–6.4)	3.4	(1.7)	(0–6.5)
Age (years)	7.5	(2.4)	(3–15.8)	7.5	(2.4)	(3–15.8)	7.6	(2.4)	(3–14.7)
BMI z-score^a^	0.4	(1.1)	(−5.4–4)	0.3	(1.2)	(−5.3–3.7)	0.4	(1.1)	(− 5.4–4.0)
Valid weartime (min / day)	695	(113)	(363–1361)	697	(117)	(366–1361)	693	(108)	(363–1334)
	*N*	%		*N*	%		*N*	%	
Moveability category									
High (score ≥ 0)	1219	49.0		623	49.1		596	48.9	
Low (score < 0)	1269	51.0		647	50.9		622	51.1	
Parental education^b^									
Missing	76	3.1		41	3.2		35	2.9	
Low (Ievel below 2)	207	8.3		106	8.4		101	8.3	
Medium (level 3, 4)	1067	42.9		542	42.7		525	43.1	
High (level 5 and more)	1138	45.7		581	45.8		557	45.7	
Parental safety concerns									
Missing	145	5.8		83	6.5		62	5.1	
No concerns	1417	57.5		723	56.9		694	57.0	
Safety concerns	926	36.5		464	36.5		462	37.9	
Sportsclub membership									
Missing	149	6.0		79	6.2		70	5.8	
Yes	1430	57.5		722	56.9		708	58.1	
No	909	36.5		469	36.9		440	36.1	
BMI categories^a^									
Normal weight	1993	80.1		1033	81.3		960	78.8	
Overweight / obese	495	19.9		237	18.7		258	21.2	
Season									
Autumn / winter	1431	57.5		725	57.1		706	58.0	
Spring / summer	1057	42.5		545	42.9		512	42.0	

^a^BMI z-score and categories according to Cole & Lobstein [25]

^b^Maximum ISCED category of both parents [26]

**Fig. 1:**
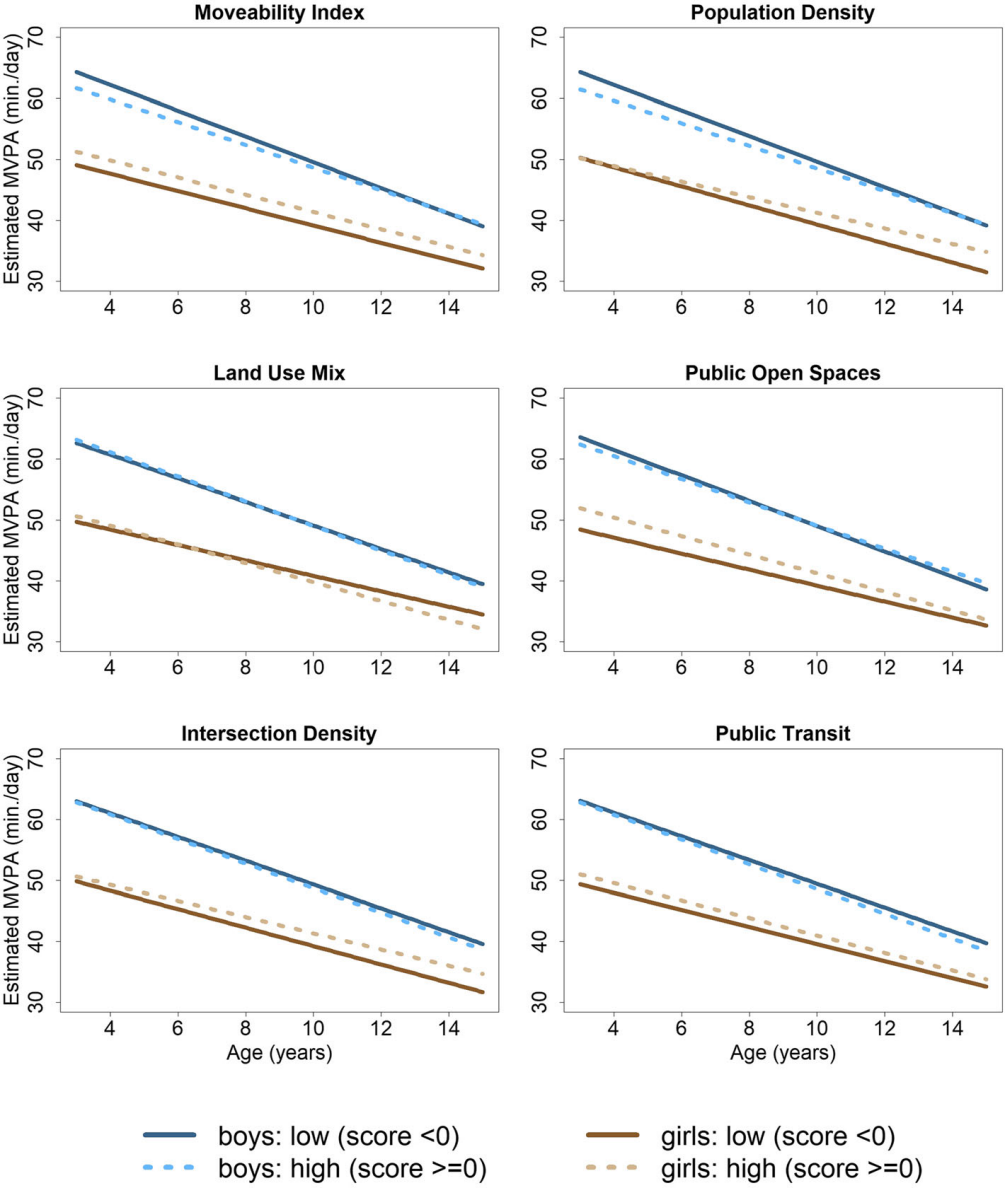
Estimated trajectories of moderate-to-vigorous physical activity (MVPA) in min./day across age (3–15 years) for boys (blue) and girls (brown) with differences of high (z-score ≥ 0; dashed lines) vs. low (z-score < 0; solid lines) in the moveability index (top-left to bottom-right) and each of its five components, i.e. residential density, land use mix, availability of public open spaces, street connectivity, and availability of public transport

**Fig. 2:**
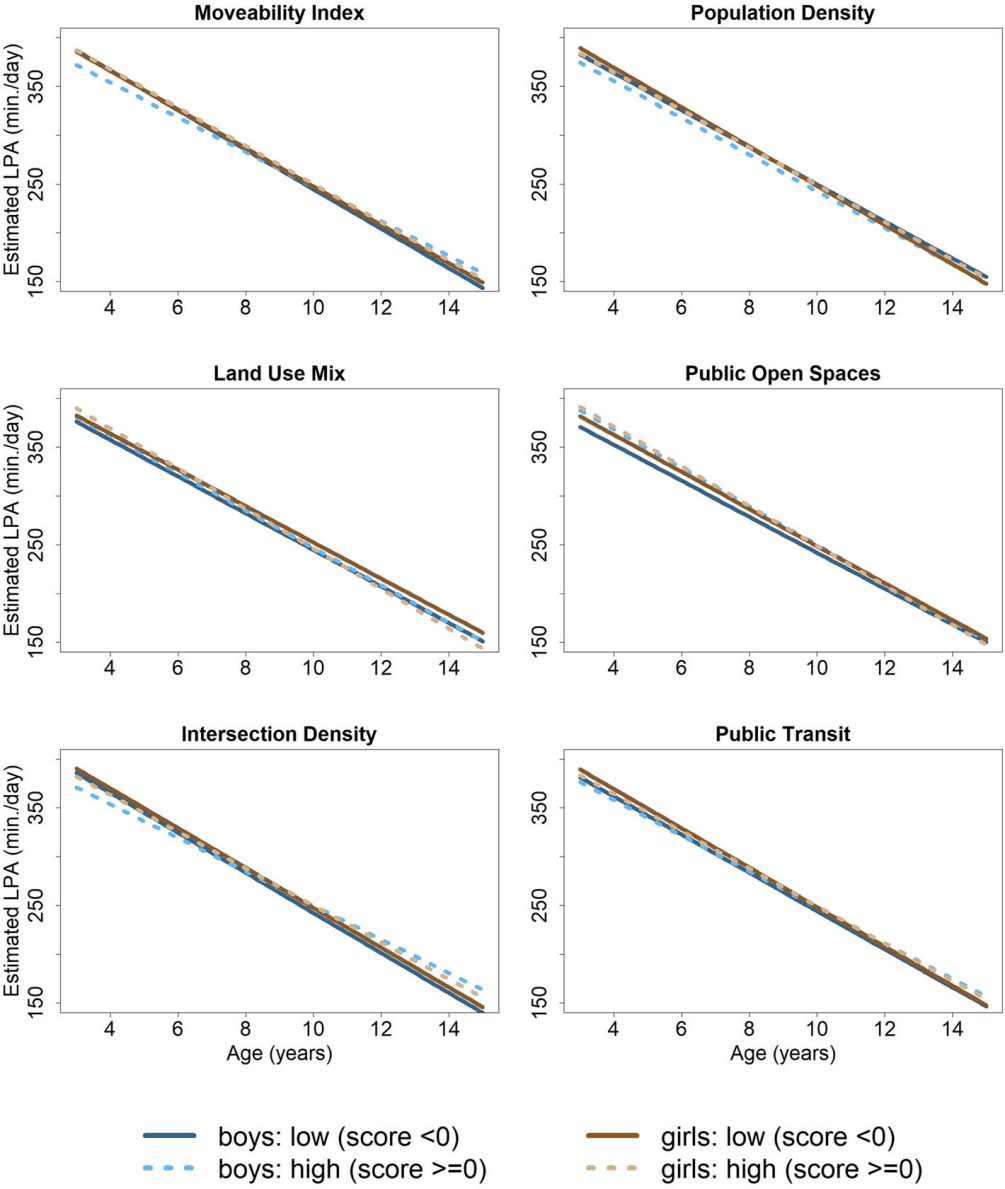
Estimated trajectories of light physical activity (LPA) in min./day across age (3–15 years) for boys (blue) and girls (brown) with differences of high (z-score ≥ 0; dashed lines) vs. low (z-score < 0; solid lines) in the moveability index (top-left to bottom-right) and each of its five components, i.e. residential density, land use mix, availability of public open spaces, street connectivity, and availability of public transport

Main and interaction effects of the linear mixed models for environmental variables and age are presented in Table 2. Parameter estimates from these models were used to estimate trajectories for MVPA (see Fig. 1) and LPA (see Fig. 2), stratified by high and low categories of environmental variables and by sex, while mean differences of these trajectories are shown for specific chosen age values are shown in Table 3. Figure 1 depicts linear trajectories of MVPA across age from 3 to 15 years estimated based on the linear mixed model. Trajectories are displayed for girls (brown) and boys (blue) living in high (dashed line) and low moveable areas (solid line). Analogously, Fig. 2 depicts estimated linear trajectories of LPA across age from 3 to 15 high vs. low categories of environmental variables and by sex.

**Table 2:** Longitudinal effects, i.e. main effects and interaction effects with age, of the moveability index and its components (high: z-score ≥ 0 vs. low: z-score < 0) on moderate-to-vigorous physical activity (MVPA) and light physical activity (LPA) for *N* = 3394 Observations of *n* = 2488 3- to 15-year old children and adolescents stratified by sex

	MVPA (min. / day)				LPA (min. / day)			
	Boys	*N* = 1717	Girls	*N* = 1677	Boys	*N* = 1717	Girls	*N* = 1677
	$\hat{\beta}$	95% CI	$\hat{\beta}$	95% CI	$\hat{\beta}$	95% CI	$\hat{\beta}$	95% CI
Moveability (high, ref.: low)	−1.30	(−3.62; 1.04)	**2.14**	**(0.11; 4.16)**	−2.54	(−8.49; 3.41)	2.21	(−4.05; 8.48)
Age	**−2.16**	**(−2.74; −1.58)**	**−1.55**	**(− 2.10; − 1.01)**	**−20.3**	**(− 21.9; − 18.6)**	**−19.8**	**(−21.4; − 18.1)**
Moveability (high, ref.: low) * age	0.20	(− 0.58; 0.97)	0.04	(− 0.71; 0.79)	**2.68**	**(0.46; 4.90)**	0.21	(−2.08; 2.49)
POS density (high, ref.: low)	−0.24	(−2.51; 2.02)	**2.38**	**(0.43; 4.34)**	**10.6**	**(4.78; 16.3)**	2.97	(−3.10; 9.03)
Age	**−2.14**	**(− 2.72; −1.55)**	**−1.46**	**(− 2.00; − 0.92)**	**− 18.3**	**(− 20.0; − 16.7)**	**− 19.1**	**(− 20.7; − 17.4)**
POS density (high, ref.: low) * age	0.15	(− 0.62; 0.92)	− 0.16	(− 0.90; 0.59)	−1.2	(− 3.43; 1.02)	− 1.24	(− 3.52; 1.04)
Residential density (high, ref.: low)	−1.52	(− 3.79; 0.75)	1.28	(− 0.70; 3.27)	**−7.82**	**(− 13.6; − 2.04)**	− 0.91	(− 7.02; 5.21)
Age	**−2.13**	**(−2.72; − 1.55)**	**−1.70**	**(− 2.24; − 1.16)**	**−19.0**	**(− 20.7; − 17.4)**	**− 20.2**	**(− 21.8; − 18.5)**
Residential density (high, ref.: low) * age	0.17	(−0.60; 0.95)	0.32	(−0.43; 1.06)	0.41	(−1.81; 2.63)	0.98	(−1.30; 3.25)
Land use mix (high, ref.: low)	0.15	(−2.16; 2.45)	−0.43	(− 2.47; 1.60)	2.67	(− 3.20; 8.54)	− 2.57	(− 8.87; 3.72)
Age	**−1.98**	**(− 2.61; − 1.34)**	**−1.40**	**(− 1.99; − 0.80)**	**− 18.8**	**(− 20.6; − 17.0)**	**− 18.6**	**(− 20.4; − 16.8)**
Land use mix (high, ref.: low) * age	−0.13	(− 0.92; 0.66)	− 0.26	(− 1.02; 0.50)	− 0.19	(− 2.45; 2.07)	−1.96	(− 4.28; 0.37)
Intersection density (high, ref.: low)	− 0.46	(− 2.76; 1.84)	1.64	(− 0.36; 3.63)	1.06	(− 4.79; 6.91)	− 0.69	(− 6.89; 5.51)
Age	**−2.01**	**(− 2.57; − 1.46)**	**−1.65**	**(− 2.18; − 1.12)**	**−20.5**	**(− 22.1; − 18.9)**	**−20.4**	**(− 22.0; − 18.8)**
Intersection density (high, ref.: low) * age	−0.09	(− 0.86; 0.68)	0.23	(− 0.52; 0.98)	**3.36**	**(1.14; 5.57)**	1.57	(−0.72; 3.85)
Public transport (high, ref.: low)	−0.65	(−3.00; 1.70)	1.39	(− 0.65; 3.43)	1.27	(− 4.73; 7.27)	− 0.94	(− 7.23; 5.35)
Age	**−2.00**	**(− 2.55; − 1.44)**	**−1.53**	**(−2.06; − 1.00)**	**−19.5**	**(− 21.1; − 17.9)**	**−20.2**	**(− 21.8; − 18.6)**
Public transport (high, ref.: low) * age	−0.13	(− 0.90; 0.64)	− 0.01	(− 0.75; 0.75)	1.38	(− 0.85; 3.61)	1.07	(−1.22; 3.35)

Linear mixed models were adjusted for maximum ISCED level of both parents, parental safety concerns, sportsclub membership, valid wear time and season of accelerometer measurements, as well as study region; effect estimates not reported

Bold significance is provided via confidence limits (significant if 0 is not included) which is similar to *p*-value

**Table 3:** Estimated mean differences and 95% confidence intervals (CI) of linear trajectories for moderate-to-vigorous physical activity (MVPA) and light physical activity () between high (high: z-score ≥ 0) and low (low: z-score < 0) categories of the moveability index and its five components at chosen age values

		MVPA (min./day)				LPA (min./day)			
		Boys	*N* = 1717	Girls	*N* = 1677	Boys	*N* = 1717	Girls	*N* = 1677
		Estimated difference	95% CI	Estimated difference	95% CI	Estimated difference	95% CI	Estimated difference	95% CI
	Age								
Moveability	4	−2.07	(− 6.30; 2.15)	1.99	(−1.85; 5.82)	**−13.3**	**(− 24.5; − 2.02)**	1.39	(− 10.8; 13.5)
(high, vs. low)	6	−1.68	(−4.72; 1.36)	2.06	(−0.62; 4.74)	**−7.90**	**(−15.7; − 0.07)**	1.80	(− 6.71; 10.3)
	8	−1.29	(− 3.62; 1.04)	**2.14**	**(0.11; 4.16)**	−2.54	(− 8.49; 3.41)	2.21	(−4.05; 8.48)
	10	−0.90	(−3.43; 1.63)	2.21	(−0.13; 4.56)	2.82	(−4.19; 9.83)	2.62	(−4.28; 9.53)
	12	−0.51	(−3.99; 2.98)	2.29	(−1.08; 5.66)	8.18	(−.193; 18.3)	3.04	(−6.85; 12.9)
	14	−0.11	(−4.88; 4.65)	2.37	(−2.29; 7.02)	13.5	(−0.41; 27.5)	3.45	**(−10.3; 17.2)**
POS density	4	−0.85	(−5.03; 3.33)	3.01	(−0.76; 6.77)	**15.3**	**(4.19; 26.5)**	7.92	(−4.04; 19.9)
(high, vs. low)	6	−0.55	(−3.53; 2.44)	**2.69**	**(0.09; 5.30)**	**12.9**	**(5.26; 20.6)**	5.44	(−2.87; 13.8)
	8	−0.24	(−2.51; 2.02)	**2.38**	**(0,43; 4.34)**	**10.5**	**(4.78; 16.3)**	2.96	(−3.10; 9.03)
	10	0.06	(−2.42; 2.54)	2.07	(−0,24; 4.38)	**8.13**	**(1.29; 15.0)**	0.49	(−6.29; 7.26)
	12	0.36	(−3.10; 3.83)	1.76	(−1.60; 5.12)	5.73	(−4.28; 15.7)	−1.99	(−11.8; 7.83)
	14	0.67	(−4.09; 5.42)	1.45	(−3.21; 6.10)	3.32	(−10.6; 17.2)	−4.47	(−18.2; 9.26)
Residential density	4	−2.21	(−6.40; 1.97)	0.01	(−3.79; 3.81)	−9.46	(−20.6; 1.69)	−4.81	(−16.8; 7.18)
(high, vs. low)	6	−1.86	(−4.85; 1.13)	0.65	(−2.00; 3.29)	**−8.64**	**(− 16.3; −0.95)**	− 2.86	(− 11.2; 5.51)
	8	−1.52	(−3.79; 0.75)	1.28	(−0.70; 3.27)	**−7.82**	**(−13.6; − 2.04)**	− 0.91	(−7.02; 5.21)
	10	− 1.17	(−3.65; 1.31)	1.92	(−0.39; 4.23)	− 7.00	(− 13.9; − 0.14)	1.05	(−5.74; 7.83)
	12	− 0.82	(−4.28; 2.64)	2.56	(− 0.79; 5.90)	−6.19	(− 16.2; 3.83)	3.00	(−6.79; 12.8)
	14	−0.47	(−5.22; 4.27)	3.19	(−1.44; 7.82)	−5.37	(−19.3; 8.52)	4.95	(−8.73; 18.7)
Land use mix	4	0.67	(−3.61; 4.94)	0.61	(−3.32; 4.55)	3.43	(−7.98; 14.8)	5.25	(−7.20; 17.7)
(high, vs. low)	6	0.41	(−2.65; 3.46)	0.09	(−2.65; 2.83)	3.05	(−4.82; 10.9)	1.34	(−7.37; 10.0)
	8	0.15	(−2.16; 2.45)	−0.43	(−2.47; 1.60)	2.67	(−3.20; 8.54)	−2.57	(−8.87; 3.72)
	10	−0.12	(−2.63; 2.40)	−0.95	(−3.29; 1.38)	2.29	(−4.62; 9.21)	−6.48	(−13.3; 0.35)
	12	−0.38	(−3.88; 3.12)	−1.48	(−4.85; 1.90)	1.92	(−8.17; 12.0)	**−10.4**	**(−20.3; −0.55)**
	14	−0.64	(−5.45; 4.18)	−2.00	(−6.69; 2.69)	1.54	(−12.5; 15.6)	**−14.3**	**(−28.1; −0.51)**
Intersection density	4	−0.11	(−4.30; 4.08)	0.71	(−3.12; 4.55)	**−12.4**	**(−23.5; − 1.22)**	−6.97	(− 19.1; 5.15)
(high, vs. low)	6	−0.29	(−3.29; 2.72)	1.17	(− 1.49; 3.84)	−5.65	(− 13.4; 2.07)	− 3.83	(− 12.3; 4.64)
	8	− 0.46	(− 2.76; 1.84)	1.63	(− 0.36; 3.63)	1.06	(− 4.79; 6.91)	− 0.69	(− 6.89; 5.51)
	10	−0.64	(− 3.15; 1.87)	2.10	(0.23; 4.42)	**7.77**	**(0.83; 14.7)**	2.45	(−4.39; 9.28)
	12	−0.81	(−4.29; 2.66)	2.56	(−0.80; 5.92)	**14.5**	**(4.41; 24.5)**	5.58	(−4.26; 15.4)
	14	−0.99	(−5.74; 3.77)	3.02	(−1.64; 7.67)	**21.2**	**(7.27; 35.1)**	8.72	*(−5.02; 22.5)*
Public transport	4	−0.13	(−4.37; 4.11)	1.40	(−2.46; 5.25)	−4.25	(−15.6; 7.05)	−5.21	(−17.3; 6.93)
(high, vs. low)	6	−0.39	(−3.45; 2.66)	1.39	(−1.30; 4.08)	−1.49	(−9.37; 6.38)	−3.07	(− 11.6; 5.44)
	8	−0.65	(−3.00; 1.70)	1.39	(−0.65; 3.43)	1.27	(−4.73; 7.27)	−0.94	(−7.23; 5.35)
	10	−0.91	(−3.46; 1.64)	1.38	(−0.98; 3.75)	4.03	(−3.03; 11.1)	1.19	(−5.75; 8.14)
	12	−1.17	(−4.68; 2.33)	1.38	(−2.01; 4.77)	6.79	(−3.38; 17.0)	3.33	(−6.61; 13.3)
	14	−1.43	(−3.21; 3.34)	1.38	(−3.31; 6.06)	9.55	(−4.48; 23.6)	5.46	(−8.35; 19.3)

Bold significance is provided via confidence limits (significant if 0 is not included) which is similar to *p*-value

MVPA significantly declined with age (Fig. 1) for both sexes but boys showed consistently higher duration in MVPA for all ages than girls. Decline in MVPA was estimated as 2 min/day ($\hat{\beta}$ = − 2.16, 95% CI: (− 2.741; − 1.58)) each year in boys and 1.5 min/day ($\hat{\beta}$ = − 1.55, 95% CI:(− 2.10; − 1.01)) each year in girls (Table 2). There were no notable differences in boys’ MVPA trajectories between high and low categories for any environmental variable (Tables 2 & 3, Fig. 1). In girls, the main effect for the moveability index showed a significantly higher MVPA of about 2 min./day ($\hat{\beta}$ =2.14, 95% CI: (0.11; 4.16), Table 2), but no substantial interaction with age. Hence, the estimated differences of girls’ MVPA trajectories (Table 3) for high moveable neighbourhoods were consistently about 2 min./day higher for all ages (1.99 (95% CI: (− 0.62; 4.74)) at age 4 up to 2.37 (95% CI: (− 2.29; 7.02)) at age 14) compared to low moveable neighbourhoods (see Fig. 1). Living in scarcely-populated neighbourhoods was associated with slightly higher MVPA in adolescent girls ($\hat{\beta}$ = 1.28, 95% CI: (− 0.70; 3.27)) compared to densely-populated neighbourhoods (see Table 2 and Fig. 1). Mean differences of MVPA trajectories increased from 0.01 min/day (95% CI: (− 3.79; 3.81)) at age 4 up to 3.19 min/day (95% CI: (− 1.44; 7.82)) at age 14 (Table 3) comparing scarcely vs. densely-populated neighbourhoods in girls. A difference in MVPA in adolescent girls was found comparing highly-connected vs. sparsely-connected neighbourhoods (intersection density, Fig. 1), where main effect and interaction effect of intersection density with age showed a small positive but non-significant effect (Table 2). Considering the mean difference of trajectories, the positive effect of intersection density increased from 0.71 min/day (95% CI: (− 3.12; 4.55)) at age 4 to 3.02 min/day (95% CI: (− 1.64; 7.67)) at age 14. In particular, MVPA was higher for all ages of girls living in areas with high availability of public open spaces ($\hat{\beta}$ = 2.38, 95% CI: (0.43; 4.34)) compared to those living in areas with low availability of public open spaces (Table 2). Mean differences of MVPA trajectories for high vs. low availability of public open spaces slightly declined from 3.01 min/day (95% CI: (− 0.76; 6.77)) at age 4 to 1.45 min/day (95% CI: (− 3.21; 6.10)) at age 14 (Table 3). With regard to land use mix and public transport, MVPA trajectories (Fig. 1) revealed no substantial differences between high and low mixed neighbourhoods as well as high or low availability of public transport (see Tables 2 & 3).

Trajectories of LPA (Fig. 2) also showed a significant age-dependent decline of about 20 min/day for each year in both boys ($\hat{\beta}$ = − 20.3, 95% CI: (− 21.9; − 18.6)) and girls ($\hat{\beta}$ = − 19.8, 95% CI: (− 21.4; − 18.1)) (Table 2). In girls, no substantial differences between high and low categories for any environmental variable were found in relation to LPA trajectory (Tables 2 & 3).

For boys living in high moveable neighbourhoods the age-dependent decline in LPA was significantly lessened ($\hat{\beta}$ = 2.68, 95%CI: (0.46; 4.90)) compared to those living in low moveable neighbourhoods. At age 14, the LSM difference was 13 min/day (95%CI: (− 0.41; 27.5)) for high vs low moveable neighbourhoods (Table 3). A similar association was found for intersection density. In boys who lived in highly-connected neighbourhoods we found a significantly positive interaction effect on the decline in LPA over age ($\hat{\beta}$ =3.36, 95% CI: (1.14; 5.57)) compared to sparsely-connected neighbourhoods (Table 2). LPA trajectories hence revealed a significant mean difference with respect to intersection density in adolescent boys at age 12 and 14 (Table 3). Residential density showed a significant negative association with LPA in boys ($\hat{\beta}$ = − 7.82, 95% CI: (− 13.6; − 2.04), Table 2). Particularly, availability of public open spaces was positively associated with LPA in boys ($\hat{\beta}$ = 10.6, 95%CI: (4.78; 16.3)) with the effect declining with age ($\hat{\beta}$ = − 1.20, 95%CI: (− 3.43; 1.02)) (see Table 2). Mean differences in LPA trajectories between high vs low availability of public open spaces were significantly positive at age 4 (15.3 min / day, 95%CI: (4.19; 26.5)) and declined to 3.32 min/day (95%CI: (− 10.6; 17.2)) at age 14. Similar to MVPA trajectories, the LPA trajectories revealed no substantial differences between categories of land use mix or public transport availability (see Tables 2 & 3).

Sensitivity analyses based on a study sample that only included participants who provided at least two observations within the cohort did not lead to substantial differences in the resulting trajecotires presented above (results not shown).

## Discussion

The longitudinal analysis of built environment characteristics in the IDEFICS/I.Family cohort identified a protective effect of certain moveability characteristics on the decrease of PA levels in the transition from childhood to adolescence. Besides the strong age-dependent decline in MVPA and LPA levels in both, boys and girls, moveability measures such as intersection density and availability of public open spaces showed positive associations with MVPA in girls and LPA in boys. Particularly in childhood, availability of public open spaces positively influenced MVPA in girls and LPA in boys, respectively. In the transition phase to adolescence intersection density positively affected the decline of MVPA in girls and of LPA in boys. While residential density showed a positive effect on MVPA trajectories in girls, we consistently found lower LPA for all ages in boys who lived in densely-populated neighbourhoods.

Between childhood and adolescence, we estimated the age-dependent decline in PA levels as about 2 min/day in MVPA for each year and 20 min/day in LPA for each year, which also matches the estimated decline in MVPA by Farooq et al. [18]. Similarly, Ortega et al. [17] found a decrease from childhood to adolescence in accelerometer-based PA levels of about 1 to 2.5 min/day in MVPA (boys and girls, respectively) and 17.6 and 14.3 min/day in LPA (boys and girls, respectively) for each year in the European Youth Heart Study.

In girls, a supportive effect of urban moveability was found with regard to MVPA, particularly for availability of public open spaces. Schipperijn et al. [9] also investigated the longitudinal effect of urban moveability on MVPA in a sample of Danish children and adolescents and found a positive effect of urban moveability on MVPA which was more pronounced in girls than in boys similar to our study. The positive effect of public open spaces, i.e. parks and playgrounds, on PA-levels has also been shown in a range of cross-sectional studies [8, 9, 27, 34, 35]. At the individual level, we used sports club membership as a covariate in our analysis, which was found to have a positive impact on overall MVPA levels in children and adolescents [36]. Besides the positive effect of public open spaces for girls at the environmental level, sports club membership had a significantly positive effect on MVPA of about 2 min/day. For boys, where no effect of public open spaces was found, sports club membership also had a positive effect on MVPA with twice the magnitude (results not presented).

While the impact of public open spaces on PA-levels declined with age, the positive effects of intersection density in adolescence on MVPA for girls and on LPA for boys increased with age. Findings in adolescents (> 12 years) for boys and girls might be explained by an increase in independent mobility in the transition phase [37] in combination with less constraints on leisure time PA and active transport through their parents [38], independent mobility has been shown to increase active transport, i.e. walking and cycling [39]. This allows adolescents to use the environment and gain similar positive effects of the street network and on active transport compared to adults [28]. Thus, the positive effect of environmental characteristics that are related to active travel in adults [40, 41] are also reflected in adolescents in our study. In contrast, young children (< 8 years) who lived in highly-connected neighbourhoods were found to show lower LPA compared to children who lived in sparsely-connected neighbourhoods. This may be due to the complexity of the street network that might hinder younger children to adhere to an active lifestyle which is strongly influenced by their parents and most likely affected by parental concerns regarding traffic safety [37, 38]. The use of the moveability index that includes availability of public open spaces might hence be limited to children, whereas in adolescents the walkability index as e.g. constructed by Freeman et al. [28] might be sufficient to investigate the built environment with regard to PA in adolescents.

The present study did not consider changes in the moveability index between a previous and a new home neighbourhood after any residential relocation of the participants. Instead, we were able to includee participants that remained over 6 years in the same neighbourhood through their transition phase from childhood to adolescence. The results imply that highly-moveable home neighbourhoods are supportive to establish a certain level of habitual PA in early childhood, particularly through the availability of sufficient spaces that encourage play and sports, while in adolescents opportunities for active transport provided by the street network become more important [39–41]. This implication is supported for example by Coombes et al. [42] who found that children who attended both a primary and secondary school with a more walkable environment were more likely to maintain active travel behaviours than those with less supportive environments. Moreover, the impact of residential relocation is mainly investigated in adults [13, 14]. With regard to the home neighbourhood of children and adolescents, more information is needed on parental self-selection and reasons why specific neighbourhood are chosen based on preferences for active transport and for practicing an active lifestyle, while the neighbourhood determines the environmental exposure to opportunities for PA [41]. Since activity patterns of parents are strongly linked to PA-levels in children, studies focussing on residential relocation need to include instruments to assess and adjust for residential self-selection [41].

Some limitations of our analyses have to be discussed. Longitudinal analyses of MVPA trajectories are based on repeated measurements, but only 5% of the study subjects provided accelerometer measurements in all three surveys. In addition the small number of repeated measurements did not allow us to model trajectories of higher complexity, which led to a lower precision in results at the edges of our age range illustrated by the large confidence limits for the mean differences in Table 3 for age 4 or 14. Further, results of the association might be affected by the spatial blurring that was implemented to use address coordinates, though only minor effects of the spatial blurring were observed in the assessment of moveability measures [29]. Parents with medium or high educational levels were overrepresented in our study and most of the accelerometer measurements took place in autumn and winter time. Both aspects might have also affected our results.

The major strength of our study is the use of a relatively large dataset incorporating repeated, objective measurements for both PA outcomes and environmental exposure of up to three surveys of the IDEFICS/I.Family cohort for some participants. Moreover, the use of linear mixed models allowed us to handle unbalanced data to create physical activity trajectories which maximised the use of participant data in analysis. We also considered the same home neighbourhood for participants in each survey. Hence, we can reduce the impact of a possible self-selection bias for the neighbourhood as well as reverse causation, since changes in PA are unlikely to have affected any changes in the built environment.

## Conclusion

Built environment characteristics that offer opportunities for active travel and leisure time PA in the home neighbourhood are important determinants of PA in children and adolescents. Particularly, in the transition from childhood to adolescence, characteristics of urban moveability revealed a supportive impact, which ameliorated the downward trajectory in PA with age. In childhood environmental support for leisure time PA through available public open spaces was found to be a protective factor whereas in adolescence highly-connected neighbourhoods were most supportive for PA.

## Data Availability

Due to the prospective nature of this ongoing cohort study, the full anonymization of study data is ruled out. Data are available on request and all requests need approval by the study’s Steering Committee. Interested researchers can contact the study co-ordinator (Ahrens@leibniz-bips.de) to request data access. All requests for accessing data of the IDEFICS/I.Family cohort are discussed on a case-by-case basis by the Steering Committee.
